# The Pro-fibrotic Response of Mesenchymal Leader Cells to Lens Wounding Involves Hyaluronic Acid, Its Receptor RHAMM, and Vimentin

**DOI:** 10.3389/fcell.2022.862423

**Published:** 2022-03-21

**Authors:** A. Sue Menko, Alison Romisher, Janice L. Walker

**Affiliations:** ^1^ Department of Pathology, Anatomy and Cell Biology, Sidney Kimmel Medical College, Thomas Jefferson University, Philadelphia, PA, United States; ^2^ Department of Ophthalmology, Sidney Kimmel Medical College, Thomas Jefferson University, Philadelphia, PA, United States

**Keywords:** lens, wound-repair, provisional matrix, hyaluronic acid, CD44, RHAMM, fibrosis

## Abstract

Hyaluronic Acid/Hyaluronan (HA) is a major component of the provisional matrix deposited by cells post-wounding with roles both in regulating cell migration to repair a wound and in promoting a fibrotic outcome to wounding. Both are mediated through its receptors CD44 and RHAMM. We now showed that HA is present in the provisional matrix assembled on the substrate surface in a lens post-cataract surgery explant wound model in which mesenchymal leader cells populate the wound edges to direct migration of the lens epithelium across the adjacent culture substrate onto which this matrix is assembled. Inhibiting HA expression with 4-MU blocked assembly of FN-EDA and collagen I by the wound-responsive mesenchymal leader cells and their migration. These cells express both the HA receptors CD44 and RHAMM. CD44 co-localized with HA at their cell-cell interfaces. RHAMM was predominant in the lamellipodial protrusions extended by the mesenchymal cells at the leading edge, and along HA fibrils organized on the substrate surface. Within a few days post-lens wounding the leader cells are induced to transition to αSMA+ myofibroblasts. Since HA/RHAMM is implicated in both cell migration and inducing fibrosis we examined the impact of blocking HA synthesis on myofibroblast emergence and discovered that it was dependent on HA. While RHAMM has not been previously linked to the intermediate filament protein vimentin, our studies with these explant cultures have shown that vimentin in the cells’ lamellipodial protrusions regulate their transition to myofibroblast. PLA studies now revealed that RHAMM was complexed with both HA and vimentin in the lamellipodial protrusions of leader cells, implicating this HA/RHAMM/vimentin complex in the regulation of leader cell function post-wounding, both in promoting cell migration and in the transition of these cells to myofibroblasts. These results increase our understanding of how the post-wounding matrix environment interacts with receptor/cytoskeletal complexes to determine whether injury outcomes are regenerative or fibrotic.

## Introduction

A tissue’s extracellular matrix (ECM) microenvironment has significant effects on cell behavior, as first revealed in studies from Mina Bissell’s lab (Bissell 2016). Their data showed that there is a dynamic reciprocity between cells and their surrounding ECM that impacts their biomechanical properties (Roskelley and Bissell 1995; Nelson and Bissell 2005; Nelson and Bissell 2006). This leads to changes in gene expression that can alter cell function to drive disease processes. More recently, it has become clear that, in response to wounding, both the composition and physical properties of the matrix produced to promote tissue repair/regeneration can become pro-fibrotic, as well as contribute to cancer progression (Lu et al., 2011; Kim et al., 2018; Basta et al., 2021; Correa-Gallegos and Rinkevich 2021; Tolg et al., 2021). These pro-fibrotic, post-wounding matrix microenvironments promote fibrosis in part by inducing cells that regulate wound-repair to acquire and maintain a myofibroblast phenotype (Hinz 2016; Karppinen et al., 2019; Tai et al., 2021).

Wound-induced provisional matrix proteins like fibronectin and collagen I are among the components of the wound-healing microenvironment that have been linked to promoting fibrosis. We show that specific expression of these matrix proteins by mesenchymal leader cells that populate the wound edge in response to cataract surgery wounding and their transition to myofibroblasts is dependent on TGFβ (Basta et al., 2021). These studies were performed with an *ex vivo* post-cataract surgery explant model that mimics both lens epithelial wound repair and the pathological fibrotic outcome of Posterior Capsule Opacification (PCO) (Walker et al., 2007; Walker et al., 2010; Menko et al., 2014; Walker et al., 2018). These wounded lens explant cultures provide an ideal reductionist model for investigating how provisional matrices organized to promote cell migration in response to wounding can lead to fibrotic disease progression. They are created by performing a mock cataract surgery on chick embryo lenses, *ex vivo*. This microsurgery removes the lens fiber cell mass and leaves behind the lens epithelial cell monolayer closely linked to the basement membrane capsule that surrounds the lens. Interdigitated among the lens epithelial cells are a subpopulation of mesenchymal cells that we have identified as resident immune cells. Resident immune cells are among the earliest responders to a wound site (Oishi and Manabe 2018), with a primary function of maintaining tissue homeostasis (Lech et al., 2012), earning them the moniker of the “sentinels of the immune system” (Davies et al., 2013). Our studies demonstrate that resident immune cells are immediate responders to lens wounding, rapidly populating the wound edges in both the chick lens mock cataract surgery explants and in human pediatric post-cataract surgery explants (Menko et al., 2021). These vimentin-rich mesenchymal leader cells express CD45, CD44 and MHCII (Walker et al., 2018; Menko et al., 2021), and display properties of professional phagocytes (Walker and Menko 2021), features they share with resident immune cells in other tissues (Arandjelovic and Ravichandran 2015; Lim et al., 2017; Oishi and Manabe 2018). Their presence in the lens and their response to lens injury involve important roles in directing lens wound repair (Menko et al., 2014; Bleaken et al., 2016). Like other tissue resident immune cells, the resident immune cells would also induce activation of an adaptive immune response, as occurs in response to lens injury (Jiang et al., 2018) and dysgenesis (Logan et al., 2017).

While the primary function of tissue resident immune cells is to maintain homeostasis, they can become agents of pathogenesis (Boyman et al., 2007; Davies et al., 2013; Richmond and Harris 2014; Ginhoux and Guilliams 2016; Lu et al., 2019; Masopust and Soerens 2019; Ardain et al., 2020). In studies with human post-cataract surgery explants we show that the CD45 ^+^ resident immune cells but not the lens epithelial cells in these explants, acquire a myofibroblast phenotype (Menko et al., 2021). Cell tracking studies using antibody to CD44 that binds exclusively to the resident immune cells identified that the resident immune cells in the chick mock cataract surgery explant cultures were the progenitors of the αSMA+ myofibroblasts that appear in the mock cataract surgery explant cultures (Walker et al., 2018). The transition to αSMA+ myofibroblasts is a hallmark of PCO, a common fibrotic pathological outcome of cataract surgery, which also is characterized by cell proliferation, migration, and deposition of matrix proteins (Wormstone et al., 2009; Wormstone and Eldred 2016; Menko et al., 2020; Walker and Menko 2021; Wormstone et al., 2021). While the matrix proteins in the wound microenvironment are key to signaling the acquisition of a fibrotic phenotype, we have also identified a requisite role for the intermediate filament protein vimentin associated with the mesenchymal leader cell population. These functions include directing the collective migration of lens epithelial cells (Menko et al., 2014), and the transition of these leader cells to a myofibroblast phenotype (Walker et al., 2018).

We now examine the impact of hyaluronic acid/hyaluronan (HA) and its receptors CD44 and Receptor for Hyaluronan Mediated Motility (RHAMM) post-wounding in studies with our clinically relevant, *ex vivo* mock cataract surgery wound repair/fibrosis model. HA is a glycosaminoglycan ubiquitously expressed in the ECM that also has been localized to the cytoplasm and the nucleus (Ripellino et al., 1988; Evanko and Wight 1999; Dicker et al., 2014). In the eye, HA is the principal component of the vitreous humor (Bremer and Rasquin 1998; Slevin et al., 2007; Theocharis et al., 2008), from where it was first purified. HA is found from early stages of development in the basement membranes of many eye tissues, including the lens (Peterson et al., 1995), and the interphotoreceptor matrix of the retina (Inatani and Tanihara 2002; Tanihara et al., 2002). A salient feature of HA is its expression in the provisional matrix that is assembled in response to tissue wounding. HA functions in promoting cell migration, a central element in modulating tissue repair and regeneration (Maytin 2016). Important to our investigations, the presence of HA in the post-wounding microenvironment is also linked to the development of fibrosis (Albeiroti et al., 2015; Maytin 2016; Tai et al., 2021). This property could, in part, reflect the reported role for HA in fibronectin fibrillogenesis (Assunção et al., 2021). HA associates either directly or indirectly with many different proteins in the ECM. The relationship between HA and the accumulation of fibronectin in the matrix environment is highlighted in studies of the trabecular meshwork that showed blocking HA synthesis reduced the presence of fibronectin (Keller et al., 2012). The complex, and sometimes antithetical, roles of HA in biological processes, pro-inflammatory and anti-inflammatory, promoting wound-repair/regeneration and promoting fibrosis, is determined by many different factors. These features include its size, modifications, structural organization, and binding to different receptors (Amorim et al., 2021).

HA synthesis and turnover are tightly regulated (Jiang et al., 2007; Aya and Stern 2014; Garantziotis and Savani 2019; Kobayashi et al., 2020). Hyaluronan synthases (HASs), are the transmembrane enzymes responsible for producing HA, while hyaluronidases are the enzymes critical to breakdown of HA (Jiang et al., 2007; Aya and Stern 2014; Garantziotis and Savani 2019; Kobayashi et al., 2020). There are three HASs; HAS1, HAS2 and HAS3, which can exhibit distinct subcellular locations, expression patterns and regulation (Itano and Kimata 2002; Heldin et al., 2019; Kobayashi et al., 2020). Typically, HASs are localized at the plasma membrane where HA is secreted to the extracellular environment (Itano and Kimata 2002; Heldin et al., 2019; Kobayashi et al., 2020).

High molecular weight (HMW) HA accumulates at sites of injury and plays roles in promoting tissue remodeling and wound repair (Jiang et al., 2007; Aya and Stern 2014; Kobayashi et al., 2020). In the wound environment, hyaluronidases and free radicals can fragment HMW-HA into low molecular weight forms (LMW-HA) that are associated with promoting inflammation and disease (Jiang et al., 2007; Aya and Stern 2014; Kobayashi et al., 2020). Many studies support a role for HA in the development of fibrosis in tissues such as the liver (Andreichenko et al., 2019; Yang et al., 2019) and lung (Li et al., 2011). HA was found to mediate fibroblast transition to a scar-inducing myofibroblast phenotype associated with fibrosis (Meran et al., 2007; Webber et al., 2009). TGFβ-induced differentiation of dermal fibroblasts to a myofibroblast phenotype is linked to HA production and assembly into a pericellular coat (Meran et al., 2007). Interestingly, in this same study, nonscarring oral fibroblasts, were found resistant to TGFβ-induced myofibroblast differentiation. This alternative response was associated with a lack of HA production and HA pericellular coat formation (Meran et al., 2007). HA synthesis is also required for TGFβ-induced differentiation of lung fibroblasts to myofibroblasts (Webber et al., 2009). Surprisingly, providing lung fibroblasts with exogenous HA blocked myofibroblast differentiation, which was associated with the relocation of the HA receptor CD44 and TGFβR ALK5 from lipid raft to non-lipid raft regions of the membrane (Webber et al., 2009). This paradoxical HA response lends support to the concept that HA presentation and organization are critical to how HA modulates cellular function (Webber et al., 2009). These studies emphasize a key role for cell-associated HA in inducing a fibrotic response, and demonstrate that HA interactions with receptors at the membrane is essential to its functional outcomes.

Principal among the many receptors for HA, are CD44 and RHAMM; others include LYVE-1, TLR2 and TLR4 (Garantziotis and Savani 2019; Abatangelo et al., 2020; Amorim et al., 2021). The interaction between HA and these receptors provide HA with the ability to activate many different downstream signaling effectors and impact cell behavior. Its cell-surface receptor CD44 is a single pass transmembrane glycoprotein expressed by a number of cell types including bone marrow mesenchymal cells, immune cells, embryonic stem cells and cancer stem cells (Levesque and Haynes 1996; Chen et al., 2018; Lee-Sayer et al., 2018). CD44 has many functions including the regulation of cell migration and cell proliferation (Krolikoski et al., 2019; Chen et al., 2020). The HA receptor RHAMM is even more complex. It is a multifunctional protein that localizes to both extracellular and intracellular sites. RHAMM has important roles both during development and in the wound-repair response that include mediating cell migration, from which it derives its name (Savani et al., 1995; Leng et al., 2019; Tolg et al., 2020). RHAMM localizes to the cell surface where it mediates binding to HA in the extracellular matrix environment, and to the cell nucleus where it associates with transcriptional complexes to regulate gene expression (Meier et al., 2014). Studies also show localization of RHAMM to the cytoplasm and its association with both microtubules and actin filaments (Assmann et al., 1999), as well as to the centrosome where it functions in regulating spindle pole stability (Maxwell et al., 2003). Following wounding, RHAMM has been specifically localized to mesenchymal cells with a fibroblastic morphology that localize to the leading edge of the wound in response to injury (Hardwick et al., 1992; Savani et al., 1995). Like their ligand HA, both CD44 and RHAMM have been linked to the development of fibrosis (Tolg et al., 2012; Cui et al., 2019; Govindaraju et al., 2019; Wu et al., 2021). In this study, we show that HA is expressed in the matrix microenvironment of the mesenchymal cells that are recruited to the leading edge of the wound where it is associated with both CD44 and RHAMM and induces cell migration and the transition of the mesenchymal leader cells to αSMA+ myofibroblasts.

## Materials and Methods

### *Ex vivo* Post-cataract Surgery Chick Explant Cultures and Inhibitor Treatment

*Ex vivo* post-cataract surgery chicken explants were created as previously described (Walker et al., 2007; Walker et al., 2010; Menko et al., 2014; Walker et al., 2018). Briefly, lenses are removed from E15 chick embryos prior to performing mock cataract surgery to remove the lens fibers cells, leaving behind the wounded epithelial cells, a population that is tightly adherent to the lens capsule basement membrane. Cuts are made in the epithelium to create a star shaped explant that is flattened on the culture substrate. Eggs are procured from Poultry Futures (Lititz, PA). Experiments using chick embryo lenses comply with ARVO guidelines for animals. All animal studies are approved by the Institutional Animal Care and Use Committee (IACUC) at Thomas Jefferson University (Philadelphia, PA). For studies blocking HA synthesis, *ex vivo* post-cataract surgery explants were treated from day (D)1 through D3 in culture with the HA inhibitor 4-Methylumbelliferone (4-MU) at 400 µM (Selleckchem, Houston, Texas (IC50: .4 mM)) or its vehicle (DMSO). Both vehicle and 4-MU were replaced each day and at D3 post-injury, cultures were fixed in 4% formaldehyde. The dose for 4-MU was chosen based both on the IC50 and the literature (Rilla et al., 2004; Vigetti et al., 2009; Saito et al., 2013). Phase contrast images were acquired with a Nikon Eclipse T*i* microscope using NIS elements software. For migration studies, time-lapse imaging was set up D1 post-injury for 24 h using a Tokai Hit stage-top incubator on a Nikon Eclipse TE2000-U microscope.

### Immunofluorescence and Live Immunolabeling

For standard immunofluorescence studies, *ex vivo* post-cataract surgery explant cultures were fixed in 4% formaldehyde for 15 min, permeabilized in 0.25% Triton-X-100 for 5 min and blocked in 5% goat serum for 30 min. Subsequently, explant cultures were incubated with primary antibodies for 30 min to 1 h followed by incubation with fluorescent-conjugated secondary antibodies (Jackson ImmunoResearch, West Grove, PA). For immunolabeling we used the following primary antibodies αSMA (Sigma Aldrich, St. Louis, MO or Abcam, Cambridge, MA), *α*-tubulin (Cell Signaling Technology, Danvers, MA), Collagen I alpha (Novus Biologicals, Centennial, CO), Fibronectin EDA (Santa Cruz Biotechnology, Santa Cruz, CA), HA (Abcam, Cambridge, MA) and RHAMM (Novus Biologicals, Centennial CO). The following primary antibodies were obtained from Developmental Studies Hybridoma Bank, created by the NICHD of the NIH (The University of Iowa, Department of Biology, Iowa City, IA): 1D10 monoclonal antibody to CD44 developed by Halfter, W.M. and AMF17B monoclonal antibody to vimentin deposited by Fulton, A.B. Explant cultures were counterstained with DAPI (Biolegend, San Diego, CA) to identify nuclei or fluorescent-conjugated Phalloidin (Invitrogen, Waltham, MA) to identify F-actin. For live antibody immunolabeling studies, cultures were incubated on the indicated day post-injury with HA (Abcam, Cambridge, MA), RHAMM (Novus Biologicals, Centennial CO), and/or CD44 antibody or with matched isotype controls (Jackson ImmunoResearch, West Grove, PA) for 20 min on ice, fixed in 4% formaldehyde and processed for immunostaining with fluorescent-conjugated secondary antibodies (Jackson Immunoresearch, West Grove, PA).

### Proximal Ligation Assay

Proximal ligation assay was performed according to manufacturer’s directions (Sigma Aldrich, St. Louis, MO) using antibodies to RHAMM and vimentin or RHAMM and HA.

### Confocal Microscopy Imaging

Images were captured using either a confocal Zeiss 510 or a confocal Zeiss 800 microscope. Z-stacks were collected with each optical plane at either 0.33 µm or 0.49 µm, as indicated. Images are shown as either single optical planes from the z-stack or as projected images.

## Results

### Emergence of Fibrotic Disease in an Ex Vivo Mock Cataract Surgery Explant Model

**FIGURE 1:**
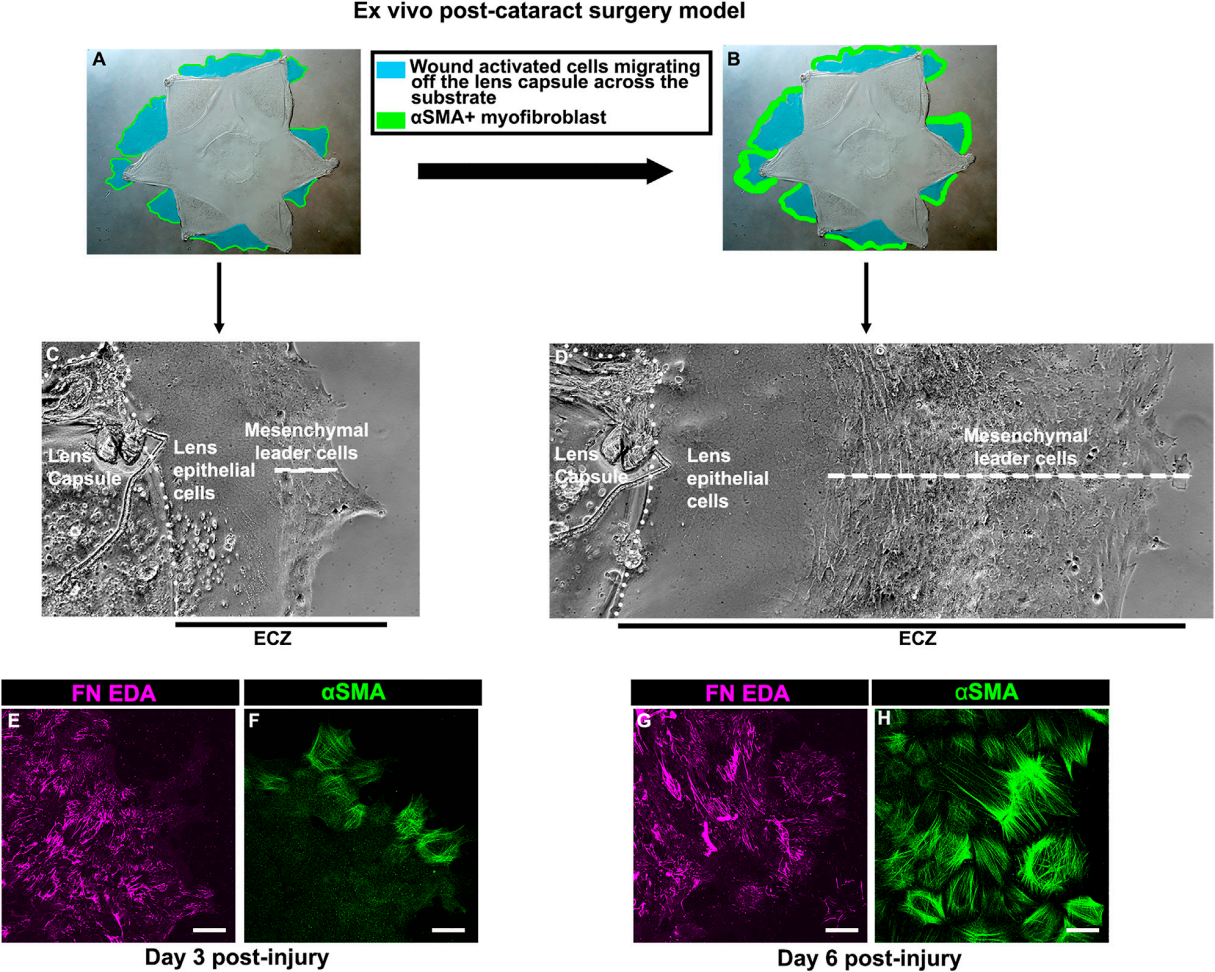
*Ex vivo* post-cataract surgery wound repair/fibrosis explant cultures. **(A,B)** Models created using a phase microscopy image of the star shaped *ex vivo* mock cataract surgery explant in culture at **(A)** culture D3 and **(B)** culture D6, on which is superimposed diagrams showing the movement of wound-activated lens epithelial cells across the adjacent culture substrate, referred to as the ExtraCapsular Zone (ECZ), shown in blue. The explant cultures also harbor a population of endogenous mesenchymal cells that rapidly migrate to the wound edges, direct the migration of the epithelium, acquire an αSMA+ myofibroblast phenotype at D3 post-wounding **(F)**, which expands greatly by D6 **(H)**, modeled in A,B in green. Phase contrast microscopy images showing the wounded lens epithelial cells and mesenchymal cells at the leading edge (white dashed line) at **(C)** D3 and **(D)** D6 post-wounding. The outside cut edge of the explant is indicated by a white dotted line. Along the substrate surface of the ECZ, the mesenchymal cells at the leading edge assemble a provisional matrix by D3 **(E)**, which expands by D6 **(G)**, which includes the ECM protein FN-EDA **(E,G)**. Images are presented as a projection from the collected confocal z-stack with each optical plane at 0.49 µm. Magnification bar = 20 µm.

To create a model that mimics conditions associated with the emergence of fibrosis post-cataract surgery we performed microsurgery *ex vivo* on isolated E15 chick embryo lenses. The procedure involves removing the differentiated fiber cells that comprise the mass of lens tissue, as in human cataract surgery (Menko et al., 2014; Walker et al., 2015). Following this, the lens epithelium, together with its subpopulation of mesenchymal resident immune cells, remain as an intact sheet closely linked to the thick basement membrane capsule surrounding the lens. The cataract-surgery wound edge is located where the fiber cells had bordered the lens epithelium. Secondary wound sites are created at cuts made in the anterior aspects of the lens capsular bag to flatten the wounded tissue explant on the tissue culture platform. From these cut sites, the wounded lens cell populations move off their basement membrane capsule onto and across the surrounding tissue culture platform (Figures 1A,B (blue)). This region is referred to as the ExtraCapsular Zone (ECZ). As the wounded lens epithelial cells migrate across the ECZ they are directed by the wound-activated mesenchymal cell subpopulation, which rapidly populates the leading edge (Figure 1C). This mesenchymal leader cell population expands as the cells migrate across the substrate (Figure 1D). On the substrate surface, the mesenchymal leader cells assemble a complex provisional matrix that, we have shown previously, includes the EDA isoform of fibronectin (FN-EDA) (Figures 1E,G). By D3 post-injury the mesenchymal leader cells express αSMA, a hallmark of their acquisition of a myofibroblast phenotype (Figure 1F). This myofibroblast population expands significantly by culture D6 (Figure 1H).

### Hyaluronic Acid Is an Integral Component of the Provisional Matrix Organized Post-lens Wounding

**FIGURE 2:**
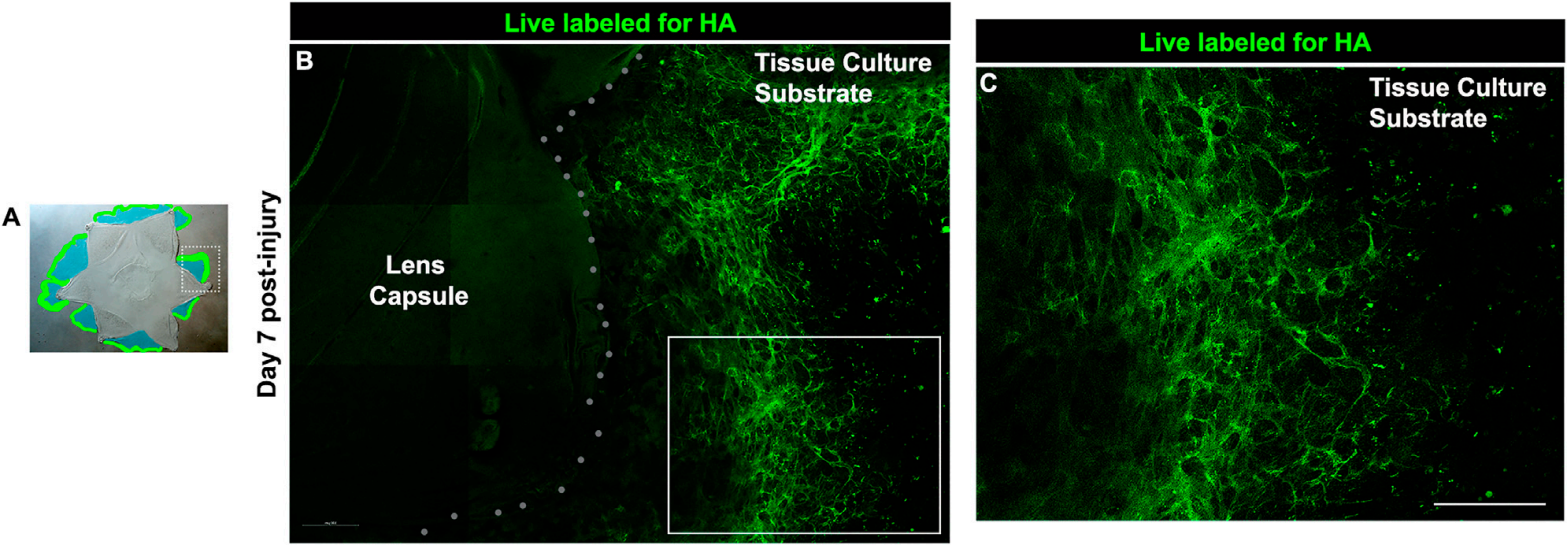
HA is a key element of the provisional matrix microenvironment post-wounding. **(A)** Post-cataract surgery explant model with a white box representative of the region examined in this study. **(B,C)** Live-immunolabeling of HA (green) of lens post-mock surgery explant cultures shown at D7 following imaging by confocal microscopy using the tiling feature to image across a large area of the ECZ. Dotted line indicates the border of the lens capsule explant and the ECZ. Boxed region in B is shown at higher magnification in C revealing that HA is synthesized and organized as a component of the provisional matrix formed in response to wounding. Magnification bar = 20 µm.

While the literature shows that hyaluronic acid (HA) is expressed in response to wounding, where it is an integral component of the provisional matrix organized to promote wound-healing, its presence in the wound microenvironment has also been linked to fibrosis (Albeiroti et al., 2015; Maytin 2016; Tai et al., 2021). We examined whether HA is a component of the provisional matrix organized on the substrate surface of the ECZ by the mesenchymal leader cells activated by mock cataract surgery wounding. For these studies, explant cultures were live-immunolabeled at 7 days post-wounding with an antibody to HA, fixed, tagged with a fluorescent-secondary antibody and imaged by confocal microscopy. The results showed that an extensive fibrillar HA network forms on the substrate surface as a component of the provisional matrix organized by the wound-activated cells migrating across the ECZ (Figure 2).

### Fibronectin-EDA Matrix Organized Along Hyaluronic Acid Fibrils Formed in Response to Lens Wounding

**FIGURE 3:**
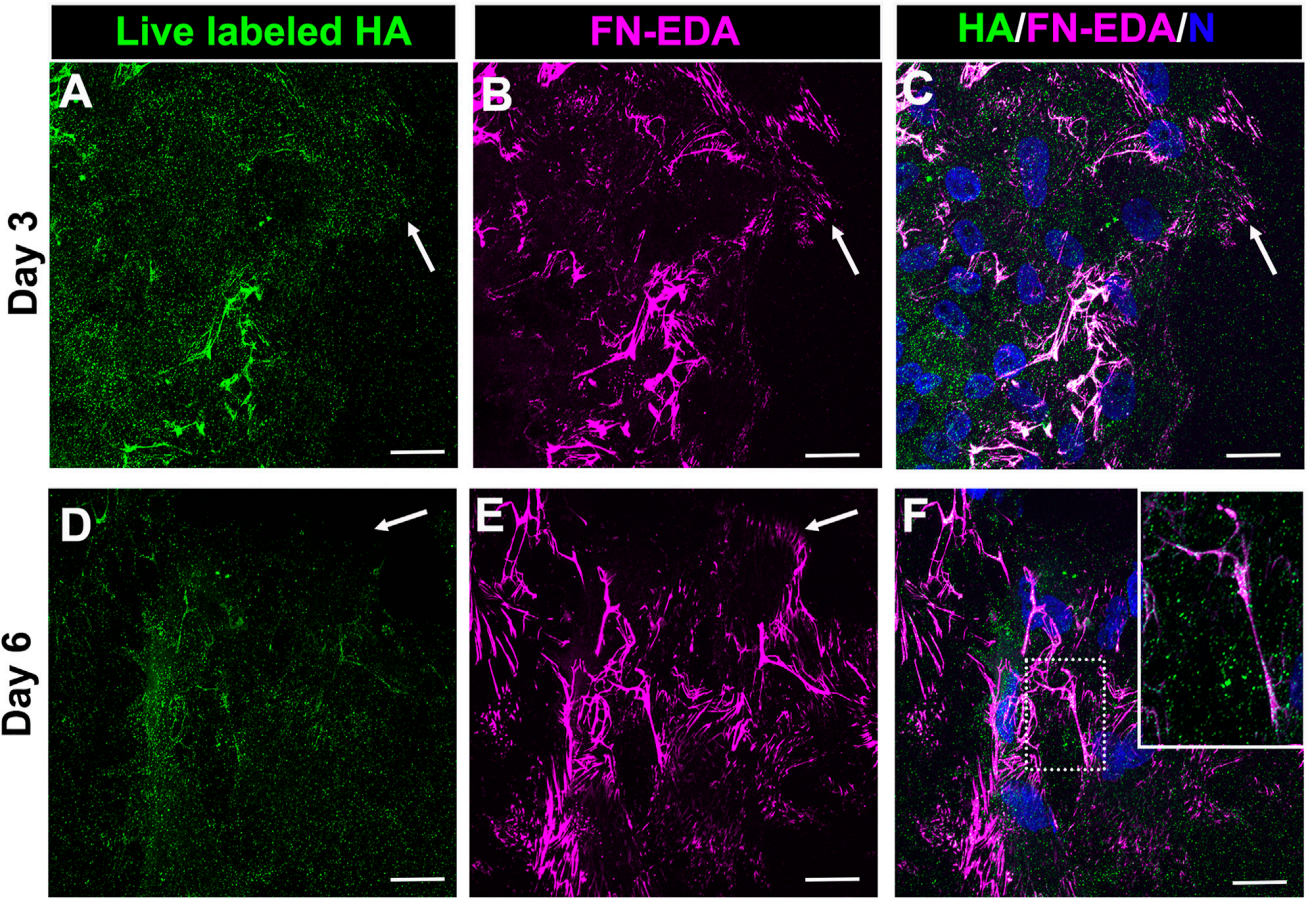
FN-EDA assembled along HA fibrils in the matrix formed post-wounding. **(A–F)** *Ex vivo* lens post-wounding explant cultures at **(A–C)** D3 and **(D–F)** D6 were live immunolabeled for HA **(A,D)**, fixed and immunolabeled for FN-EDA **(B,E)** and imaged at the leading edge of the ECZ (indicated by white arrow) by confocal microscopy. **(C,F)** Colocalization of HA and FN-EDA shown together with DAPI labeling of nuclei. Box with white dotted line in F is shown as an inset at higher magnificent and intensity adjusted. These studies show that FN-EDA is organized along HA fibrils. Images are presented as a projection from the collected confocal z-stack with each optical plane at 0.49 µm. Magnification bar = 20 µm.

As we have shown previously (Basta et al., 2021), a FN-EDA provisional matrix is organized at the leading edge of the wound-activated cells migrating across the substrate surface adjacent to the injured lens explant (Figures 1E,G). We now investigated whether the HA provisional matrix produced by these cells in response to mock cataract surgery wounding provides a substrate on which FN-EDA fibrils or organized. Mock cataract surgery explant cultures were live labeled for HA at both 3- and 6-days post-wounding. Following fixation and tagging with secondary antibody, the cultures were co-immunolabeled with an FN-EDA antibody, and imaged by confocal microscopy (Figure 3). These studies revealed that the FN-EDA matrix assembled on the substrate at the leading edge of the ECZ during the first 3 days post-wounding is organized along the fibrils of the provisional HA matrix produced by the wound-activated mesenchymal leader cells (Figures 3A–C). The coincidence of the HA and FN-EDA matrices on the substrate surface beneath the mesenchymal cell population at the leading edge of the extracapsular zone remains a defining feature of the explant cultures at culture day 6 (Figures 3D–F).

### Hyaluronic Acid Receptors CD44 and Receptor for Hyaluronan Mediated Motility Expressed by Leading Edge Cells Post-lens Wounding

**FIGURE 4:**
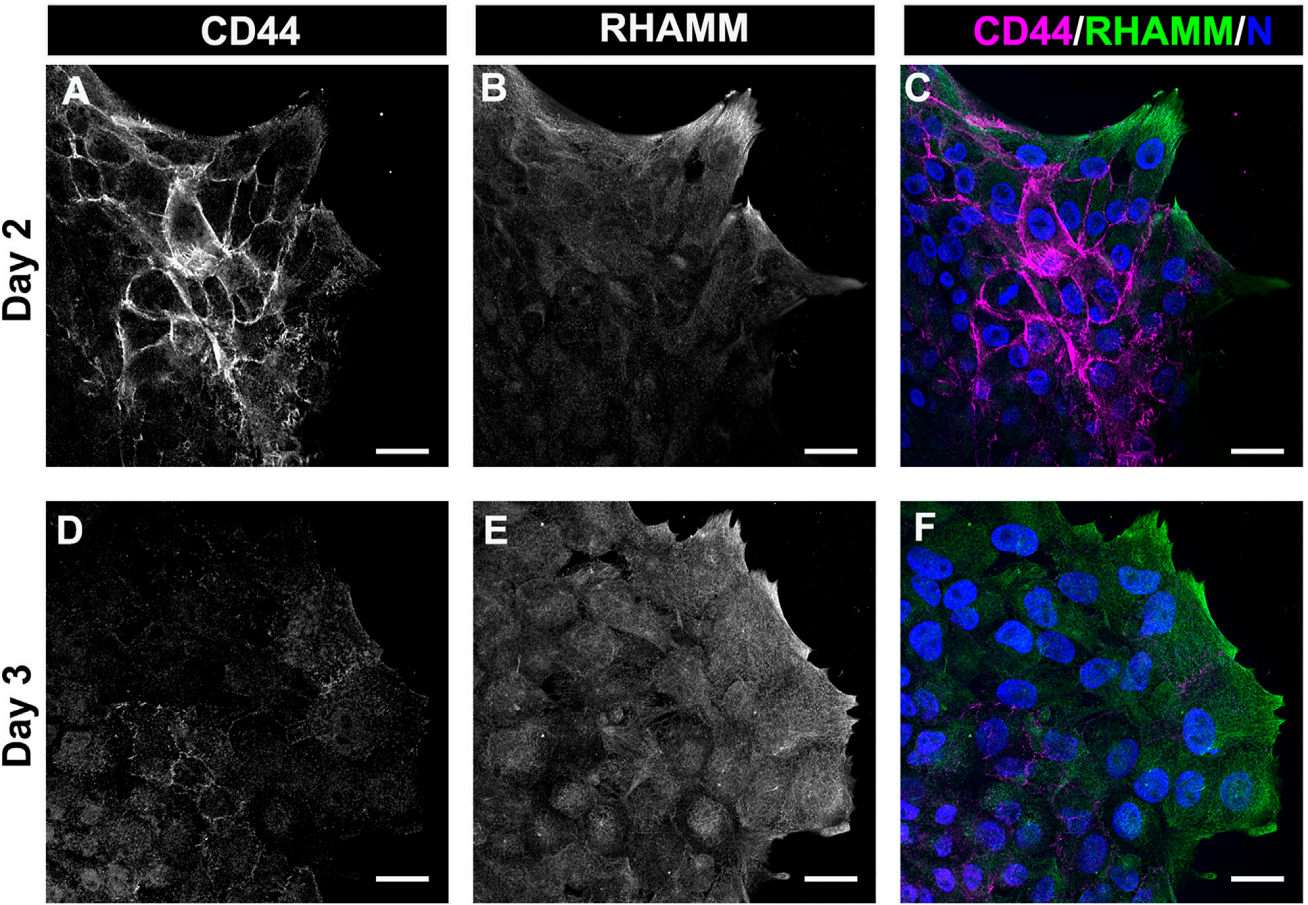
The HA receptors CD44 and RHAMM are expressed by mesenchymal leader cells responding to lens wounding. *Ex vivo* lens post-wounding explant cultures at **(A–C)** D2 and **(D–F)** D3 co-immunolabeled for **(A,D)** CD44 and **(B,E)** RHAMM, imaged by confocal microscopy at the leading edge of the ECZ, and **(C,F)** shown as merged images together with DAPI labeling of nuclei. CD44 and RHAMM have distinct patterns of localization, with CD44 most prominent at the edges of the mesenchymal cells along their cell-cell borders and RHAMM predominately localized to the lamellipodial processes extended along the substrate at the leading edge, co-localizing with CD44 at the tips of these lamellipodia. Images are presented as single optical section from a confocal z-stack with each optical plane at 0.49 µm. Magnification bars = 20 µm.

The most prominent among the HA receptors are CD44 and RHAMM. They are molecularly distinct receptors. CD44 is a transmembrane glycoprotein typically expressed by immune cells, bone marrow mesenchymal cells, embryonic stem cells, and cancer stem cells (Chen et al., 2018). RHAMM is commonly expressed by the migrating cells at a wound edge with multiple subcellular regions of localization including the cell surface, the cytoplasm, and the nucleus (Hardwick et al., 1992; Savani et al., 1995). In previous studies, we had identified that CD44 was expressed by the mesenchymal cells that populate that wound edge in response to mock cataract surgery (Walker et al., 2018; Basta et al., 2021). We now have performed a co-localization analyses to determine the relative patterns of expression of CD44 and RHAMM by the mesenchymal cells at the leading edge of the ECZ during the first few days following mock cataract surgery wounding. For these studies, the wounded lens explant cultures were fixed and permeabilized at culture D2 and D3, prior to immunolabeling for CD44 and RHAMM. The results showed that both these HA receptors were expressed by the cells that populate the leading-edge post-mock cataract surgery wounding, each with a distinct pattern of expression (Figure 4). At D2 post-wounding, CD44 was most highly expressed along their cell borders and was also localized to the tips of the lamellipodial processes they extend along the substrate at the leading edge (Figures 4A,C). While labeling intensity was diminished, this pattern of CD44 localization was retained at culture day 3 when these cells first acquire a myofibroblast phenotype (Figures 4D,F). In contrast to CD44, RHAMM was primarily localized to the lamellipodial processes extended by the mesenchymal cells at the leading edge of the ECZ at culture day 2 (Figure 4B). Here, there is some co-localization of RHAMM with CD44 at the cells’ lamellipodial tips (Figure 4C). This pattern of RHAMM localization is retained by the cells at the leading edge at D3 post-wounding, after these cells have acquired a myofibroblast phenotype (Figures 4E,F).

### CD44 Colocalizes With Hyaluronic Acid at Cell-Cell Interfaces of Leader Cells Post-wounding

**FIGURE 5:**
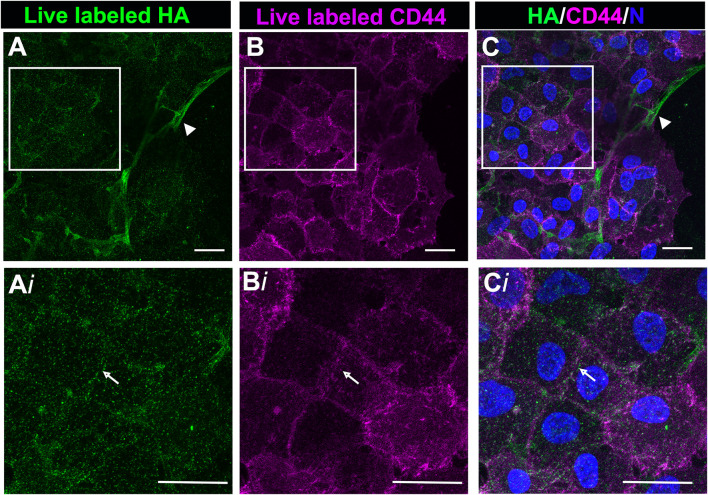
CD44 colocalized with HA in the region of cell-cell interfaces of mesenchymal leader cells. *Ex vivo* post-cataract surgery explant cultures were live immunolabeled at D7 for both HA **(A,Ai)** and CD44 **(B,Bi)**, imaged by confocal microscopy at the leading edge of the ECZ and **(C,Ci)** shown as merged images together with DAPI labeling of nuclei. Boxed areas in A-C are shown at higher magnification in Ai-Ci. CD44 colocalizes with HA along cell-cell interfaces (white arrow). No labeling for CD44 was detected along HA fibrils (arrowhead). Images are presented as a projection from a collected confocal z-stack with each optical plane at 0.49 µm. Magnification bars = 20 µm.

Following their transition to myofibroblasts, these cells expand to comprise a large area at the leading edge of the ECZ (Figures 1D,H). The coincidence of HA with its receptor CD44 was investigated in these cells by live labeling the mock cataract surgery explant cultures at 7 days post-wounding for both HA and CD44. High-resolution confocal microscopy imaging was performed at the leading edge of the ECZ (Figure 5). This approach revealed two distinct patterns of HA organization in the matrix microenvironment, one aligned with cell-cell borders (Figure 5A, arrow, from boxed in region of Figure 5A), the other organized in fibrous cords (Figure 5A, arrowhead). CD44 was colocalized with HA at cells’ lamellipodia edges concentrated along their cell-cell borders (Figure 5B,Bi,C,Ci). No labeling for CD44 was observed along the network of HA extracellular matrix fibrils (Figures 5A,C, arrowhead).

### Receptor for Hyaluronan Mediated Motility Colocalizes With Hyaluronic Acid Fibrillar Network Post-wounding

**FIGURE 6:**
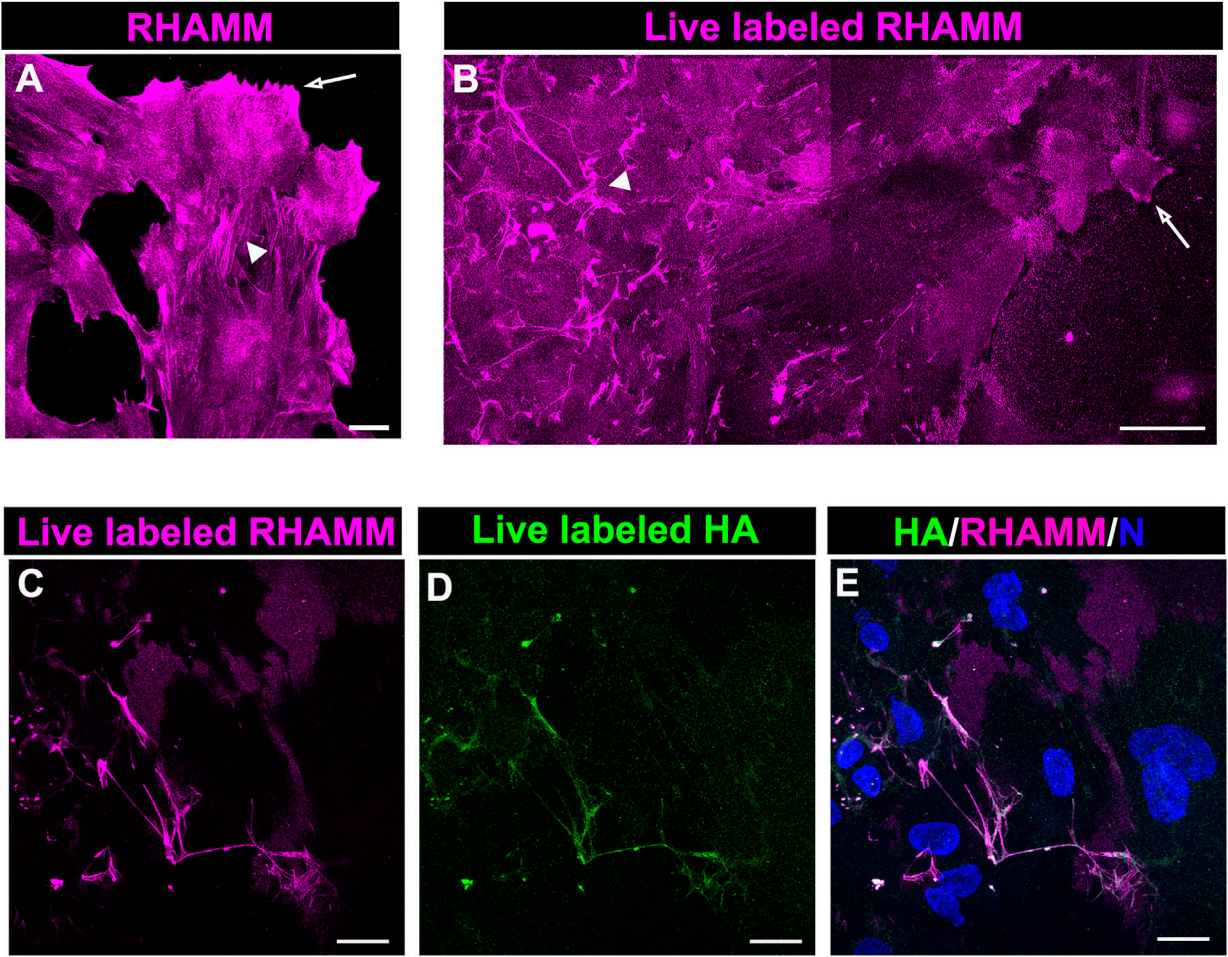
RHAMM localizes along HA fibrils. To distinguish intracellular and extracellular RHAMM populations, *ex vivo* post-cataract surgery explant cultures at D6 were either **(A)** fixed, permeabilized, and immunolabeled for RHAMM or **(B)** live-labeled with the RHAMM antibody and imaged at the leading edge of the ECZ by confocal microscopy. These approaches revealed the presence of RHAMM **(A)** along cytoskeletal-structures (arrowhead) and **(A,B)** in the cells’ lamellipodial processes (arrow), as well as **(B)** as a cell-surface associated population with a fibrillar organization (arrowhead). Co-localization of cell surface of RHAMM with HA fibrils was demonstrated by live immunolabeling at D6 for both **(C)** RHAMM and **(D)** HA, imaged by confocal microscopy at the leading edge of the ECZ and **(E)** shown as merged images together with DAPI labeling of nuclei. **(A,C-E)** are projections created from collected confocal z-stacks with each optical plane at 0.49 µm, **(B)** is a tiled confocal image. Magnification bars = **(A,C-E)** 20µm; **(E)** 50 µm.

The HA receptor RHAMM, which functions in cell migration, has roles as both a cell surface and an intracellular molecule. To provide insight into the different RHAMM localizations in the migrating cells at the leading edge of the ECZ, explant cultures at 6 days post-wounding wounding were either fixed and immunolabeled for RHAMM (Figure 6A) or live labeled with the RHAMM antibody (Figure 6B). Confocal microscopy imaging at the leading edge revealed the presence of both intracellular (Figure 6A) and cell surface-associated (Figure 6B) populations of RHAMM at D6 post-wounding. In the fixed explant cultures, RHAMM localized to the tips of the lamellipodia that the cells extended along the cell surface (Figure 6A, arrow). This pattern of localization was consistent with that observed for RHAMM in leading edge cells at culture D2 and D3 (Figures 4B,E). At D6, RHAMM also localized along actin stress fiber-like cytoskeletal structures in this mesenchymal leader cell population (Figure 6A, arrowhead). Live labeling revealed that cell-surface linked RHAMM was present at the tips of the protrusions the cells extend at the leading edge (Figure 6B, arrow) and in a filamentous distribution in the cells just behind the migrating edge with a distribution similar to that of the fibrous network of HA (Figure 6B, arrowhead). To examine the co-incidence of this cell surface RHAMM population with the HA extracellular fibrils *ex vivo* post-cataract surgery explants were live labelled with antibodies to both RHAMM and HA. Confocal imaging at the leading edge of the ECZ confirmed the colocalization of cell-surface RHAMM with HA fibrils (Figures 6C–E).

### Hyaluronic Acid and Receptor for Hyaluronan Mediated Motility Co-localize Along αSMA+ Stress Fibers in Myofibroblasts That Emerge Post-wounding

**FIGURE 7:**
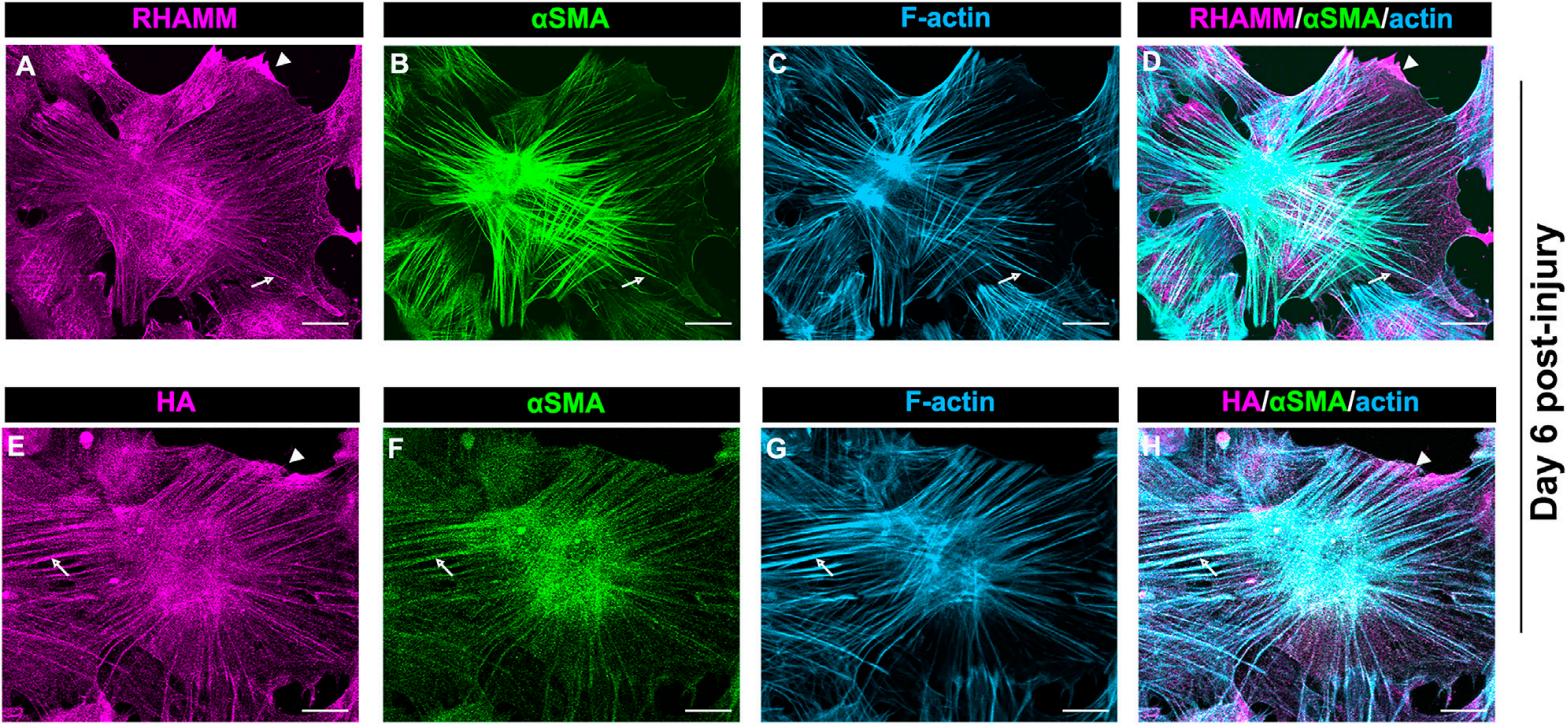
HA/RHAMM localize along αSMA+ stress fibers of myofibroblasts. *Ex vivo* post-cataract surgery explant cultures at D6 were immunolabeled for either **(A)** RHAMM or **(E)** HA and each immunolabeled for **(B,F)** αSMA, and **(C,G)** colabeled for F-actin with fluorescent conjugated phalloidin and imaged by confocal microscopy. **(D)** Merged image of RHAMM, αSMA and F-actin; **(H)** Merged image of HA, αSMA and F-actin. RHAMM and HA both were co-localize along αSMA+ stress fibers and were coincident with F-actin (arrows). HA and RHAMM were also localized to lamellipodial protrusions at the leading edge (arrowheads). All images are presented as projections from collected confocal z-stacks with each optical plane at 0.49 µm. Magnification bars = 20 µm.

The localization of intracellular RHAMM in mesenchymal leader cells that have acquired a myofibroblast phenotype at D6 resembles that of actin stress fibers, suggesting its association with these cytoskeletal filaments (Figure 6A). As previous studies provide evidence that RHAMM can interact with actin filaments (Assmann et al., 1999), we examined whether RHAMM function in myofibroblasts may involve its cooperation with αSMA+ stress fibers. For these studies, *ex vivo* mock cataract surgery cultures were fixed and permeabilized at D6 post-wounding, co-immunolabeled for αSMA and RHAMM, and labeled with a fluorescent-conjugated phalloidin to detect F-actin. Imaging was performed by confocal microscopy at the leading edge of the ECZ. The results confirm that RHAMM is localized along αSMA+ stress fibers and co-incident with F-actin (Figures 7A–D, arrows). In these same cells, RHAMM is also found in the tips of the lamellipodial protrusions extended by the myofibroblasts along the substrate at the leading edge (Figures 7A,D arrowhead).

Since HA can interact with RHAMM both outside and inside the cell (Hascall et al., 2004), we investigated whether HA also localizes along αSMA+ stress fibers in the myofibroblasts at the leading edge of the ECZ at D6 post-wounding. For these studies, the wounded explant cultures were fixed and permeabilized prior to labeling for F-actin, αSMA and HA. Similar to RHAMM (Figures 7A–D), HA was localized along αSMA+ stress fibers (Figures 7E–H, arrows), as well as to lamellipodial protrusions of the myofibroblasts at the leading edge (Figures 7E,H, arrowhead). These findings suggest that HA/RHAMM may have a role in regulating the contractile machinery of myofibroblasts.

Another cytoskeletal structure that RHAMM has been shown to associate with are the microtubules, an association that is both context-specific and cell cycle-dependent, often involving its presence at the spindle poles (Assmann et al., 1999; Tolg et al., 2010; Chen et al., 2014). *Ex vivo* cataract surgery explant cultures co-immunolabeled for *α*-tubulin and RHAMM at D2 and D3 post-wounding and imaged by confocal microscopy at the leading edge of the ECZ showed that while these cells have an extensive microtubule network there was no evidence of their co-localization with RHAMM (Supplementary Figure S1). The most prominent localization of RHAMM in these cells was to their lamellipodia protrusions.

### Novel Association of Receptor for Hyaluronan Mediated Motility With Vimentin in Leader Cell Lamellipodial Protrusions Post-wounding

**FIGURE 8:**
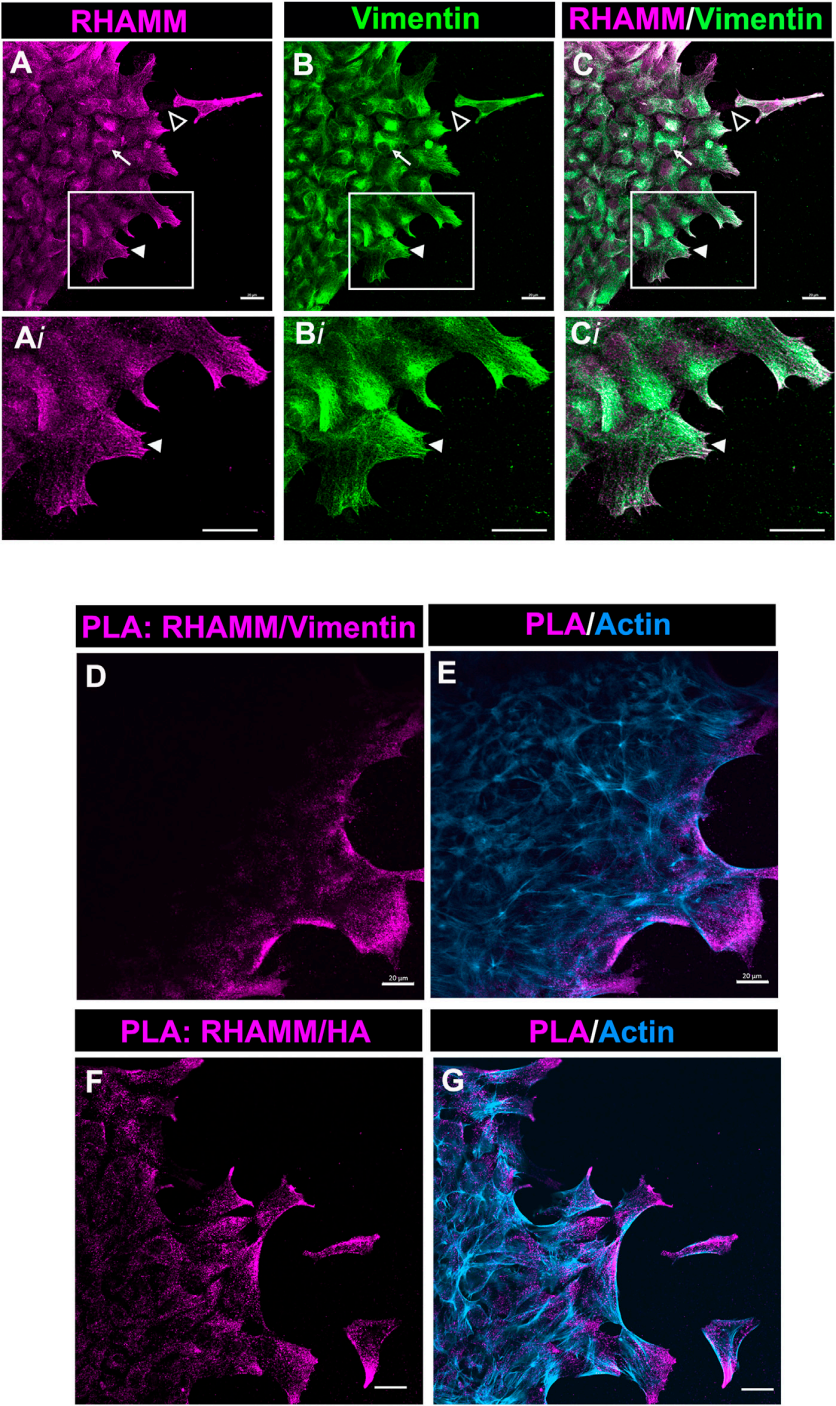
RHAMM associates with vimentin at lamellipodial protrusions extended by mesenchymal leader cells. **(A–C)** *Ex vivo* lens post-wounding explant cultures at D2 were co-immunolabeled for **(A)** RHAMM and **(B)** vimentin, imaged by confocal microscopy at the leading edge of the ECZ, and **(C)** shown as a merged image. Boxed regions in **(A–C)** are shown at higher magnification in **(A*i*-C*i*)**. The images show significant co-localization of RHAMM and vimentin in the lamellipodial extensions (arrowhead) of the mesenchymal cells at the leading edge of the ECZ. RHAMM was also co-localized with to the vimentin cytoskeletal network (arrow) and detected as puncta along the substrate (open arrowhead). **(D,E)** PLA was performed at D2 post-mock cataract surgery wounding for **(D)** RHAMM and vimentin or **(F)** HA and vimentin to determine if there was a near-neighbor association (within 40 nm or less) between these molecules and imaged by confocal microscopy at the leading edge of the ECZ. **(E,G)** shows the PLA finding together with co-labeling for F-actin. The results provides evidence of a complex consisting of HA, RHAMM and vimentin in leader cell lamellipodial protrusions. All images are presented as projections from collected confocal z-stacks with each optical plane at 0.49 µm. Magnification bars = 20 µm.

Our studies show that in the mesenchymal cells at the leading edge of the ECZ post-cataract surgery wounding RHAMM was consistently localized to the tips of lamellipodial protrusions (Figures 4B,E, Figures 6A, 7A). This pattern of localization is similar to that of the intermediate filament protein vimentin shown in our previous studies (Walker et al., 2018). Importantly, we had discovered that blocking vimentin function impairs cell migration post-wounding and blocks the transition of mesenchymal leader cells to myofibroblasts (Menko et al., 2014; Walker et al., 2018). We investigated whether RHAMM could associate with vimentin at these leader cell lamellipodial protrusions. *Ex vivo* mock cataract surgery explant cultures were co-labeled for RHAMM and vimentin at D2 post-wounding and imaged at the leading edge of the ECZ by confocal microscopy. These studies revealed that RHAMM and vimentin were highly co-localized at the lamellipodial extensions extended along the substrate surface at the leading edge of the ECZ (Figures 8A–C, 8Ai-Ci, arrowhead). Co-localization was also observed along the vimentin cytoskeletal network (Figure 8C, arrow). In addition, there were regions where punctate labeling of RHAMM was localized to the substrate surface (Figure 8A, open arrowhead, and Supplementary Figure S2A, Ai).

To further investigate this discovery that RHAMM is colocalized with vimentin, we used the Proximal Ligation Assay (PLA), an *in-situ* assay that identifies protein-protein interactions within 40 nm. PLA was performed on the explant cultures at D2 post-wounding with antibodies to vimentin and RHAMM, and the cultures post-labeled for F-actin. The results revealed that there is a close association between RHAMM and vimentin at the lamellipodial protrusions of cells at the leading edge of the ECZ (Figures 8D,E). These are the first studies that link RHAMM function to the vimentin intermediate filament cytoskeleton. Since HA was also found enriched within the membrane protrusions of the cells at the leading edge of the ECZ (Figure 7E), we examined the potential link between HA and RHAMM by PLA. The results also showed a close association between RHAMM and HA that is specific to the cell protrusions of the cells at the leading edge of the ECZ (Figures 8F,G). These were the same sites as there is a positive PLA signal for RHAMM and vimentin. These findings suggest that the lamellipodial protrusions of the leader cells are enriched with a HA/RHAMM/vimentin complex. Since our previous studies showed that vimentin plays a role in mediating leader cell transition to a myofibroblast phenotype post-mock cataract surgery wounding (Walker et al., 2018), our new findings suggest that this function is likely to be coordinated with HA/RHAMM.

### Blocking Hyaluronic Acid Synthesis Prevents Leader Cell Production of a Pro-fibrotic Provisional Matrix, Suppresses Their Migration and Blocks Their Transition to an αSMA+ Myofibroblast Phenotype

**FIGURE 9:**
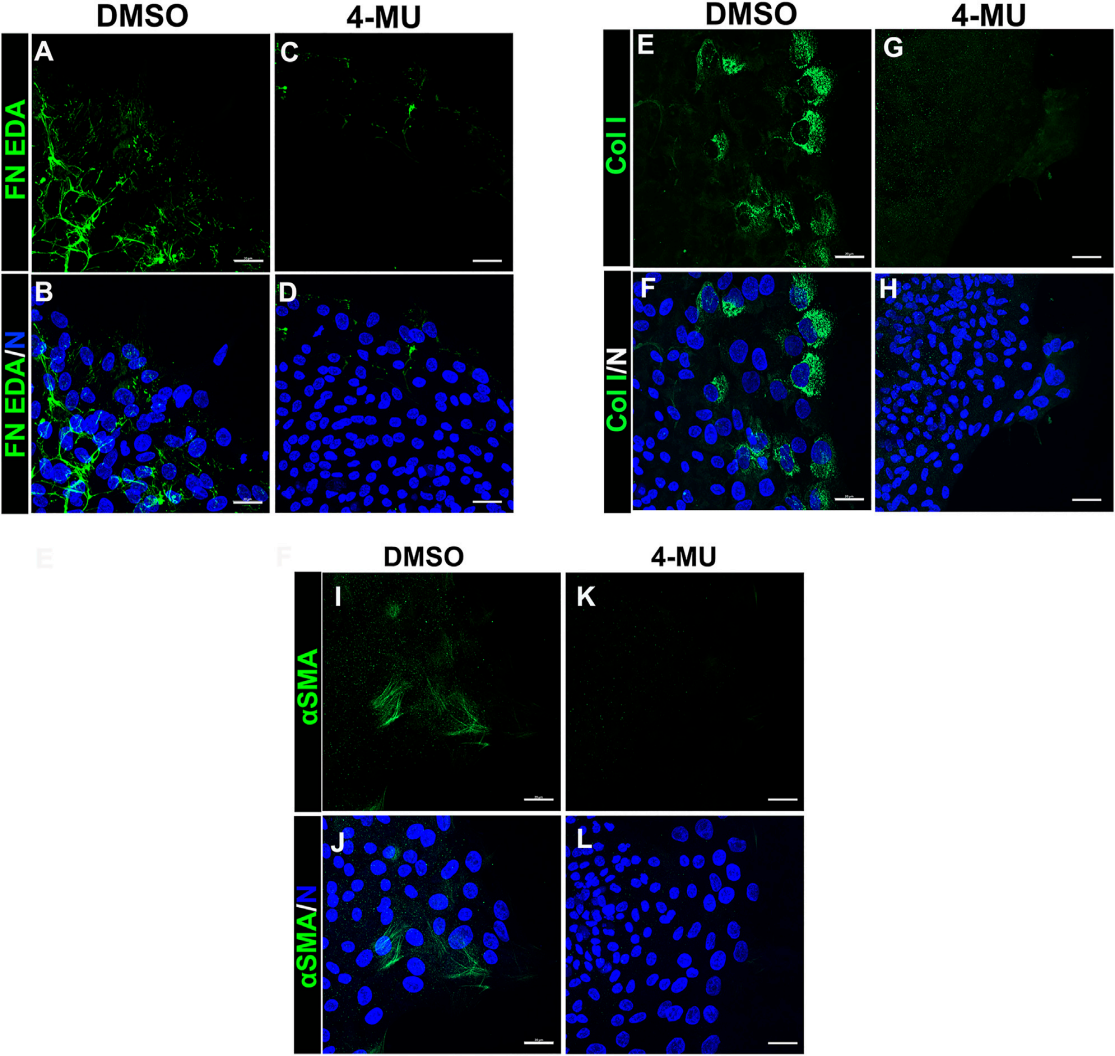
Blocking HA expression with 4-MU prevents assembly of a FN-EDA, collagen I rich provisional matrix and the transition of mesenchymal leader cells to a myofibroblast phenotype in response to wounding. **(A–L)** *Ex vivo* cataract surgery explants were treated in culture from D1-D3 post-wounding with **(C,D,G,H,K,L)** the HA synthesis inhibitor 4-MU (400 µM) or **(A,B,E,F,I,J)** its vehicle DMSO, immunolabeled for **(A–D)** FN-EDA **(E–H)** collagen I or **(I–L)** αSMA, co-labeled with DAPI, and imaged by confocal microscopy at the leading edge of the ECZ. Blocking expression of HA with 4-MU prevented leader cell assembly of a FN-EDA and Collagen I rich ECM microenvironment and prevented the emergence of αSMA+ myofibroblasts. Images are presented as projections from collected confocal z-stacks with each optical plane at 0.33 µm. Magnification bars = 20 µm. The data presented represents at least 3 independent studies.

**FIGURE 10:**
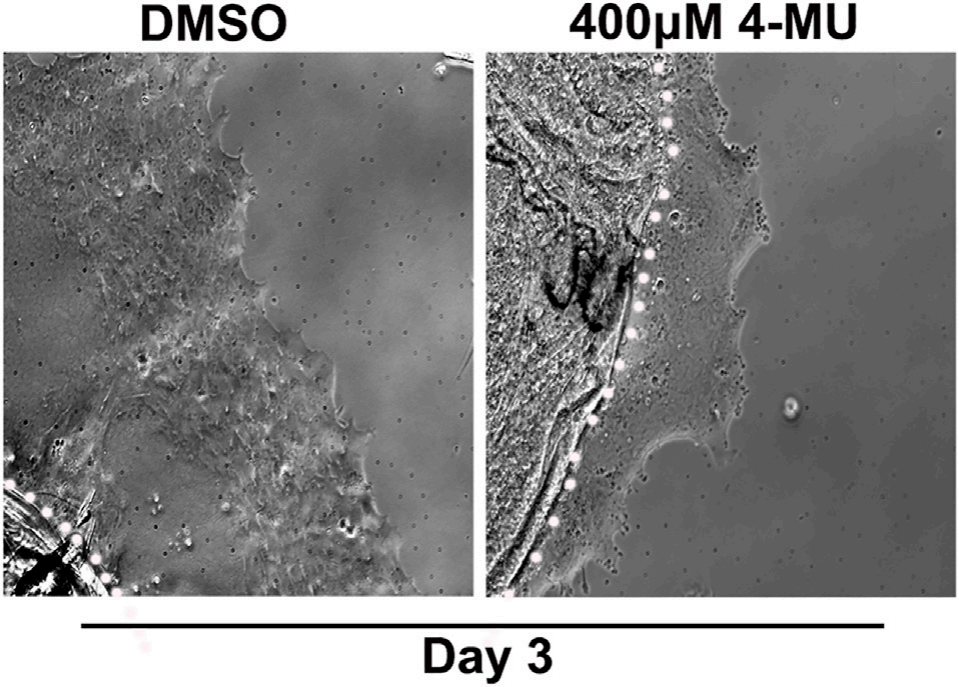
Blocking HA synthesis inhibits migration of cells within the ECZ. Ex vivo post-cataract surgery explants were treated in culture from D1-D3 post-wounding with the HA synthesis inhibitor 4-MU (400 µM) or its vehicle DMSO. Phase images of the ECZ were captured 48hrs post-treatment on D3, revealing that 4-MU treatment reduced cell migration in the ECZ region compared to vehicle controls. White dotted line marks the border between the explant and the ECZ.

Our findings showed that HA is an integral component of the FN-EDA/collagen I provisional matrix that is organized by the mesenchymal leader cells as they migrate across the ECZ in response to lens wounding. Here, they express RHAMM and CD44 with different patterns of localization, each of which co-localizes with HA. In addition to promoting processes like migration that are essential to wound repair, microenvironments rich in FN-EDA and collagen I are also considered pro-fibrotic (Muro et al., 2008; Bhattacharyya et al., 2014; Herrera et al., 2018). Their organization in a wound environment has been linked to the presence of HA (Assunção et al., 2021). Therefore, we performed functional studies to determine the impact of the HA synthesis inhibitor 4-MU on the assembly of these matrix proteins in the ECZ, the migration of the mesenchymal leader cells and their associated lens epithelium across the ECZ and the appearance of myofibroblasts at the leading edge. For these studies, wounded lens mock cataract surgery explant cultures were exposed to the HA synthesis inhibitor 4-MU or the vehicle DMSO from D1 through D3 post-injury. D3 is the time when the mesenchymal cells at the leading edge have acquired a myofibroblast phenotype. Confocal microscopy imaging was performed following immunolabeling for FN-EDA (Figures 9A–D), collagen I (with an antibody that recognizes both pro-collagen I and collagen I) (Figures 9E–H) and αSMA+ (Figures 9I–L). The results showed that blocking HA synthesis with 4-MU prevented the assembly of both FN-EDA and collagen I matrices in the extracellular microenvironment of the mesenchymal leader cells. Blocking HA expression also blocked the transition of the mesenchymal leader cells to an αSMA+ myofibroblast phenotype. The prevention of this key hallmark of fibrosis is likely the direct result of the failure to assemble a profibrotic ECM. As HA/RHAMM also has key roles in promoting cell migration post-wounding, we examined the impact of the absence of this provisional matrix microenvironment in the ECZ microenvironment. The wounded explant cultures were exposed to the HA synthesis inhibitor 4-MU or their vehicle DMSO from D1 through D3 post-injury and examined by phase contrast microscopy (Figure 10), and with time-lapse microscopy imaging (Supplementary Movies S1, S2). Blocking HA synthesis greatly suppressed but did not block cell migration across the ECZ. These results suggest the HA plays an essential role in the formation of the provisional matrix in response to cataract surgery wounding that is required for both the promotion of cell migration and the development of fibrosis.

## Discussion

A provisional matrix is formed in the wound microenvironment to promote cell migration across the injured area and close the wound (Barker and Engler 2017; Chester and Brown 2017; Johnson et al., 2018; McKay et al., 2019; Das et al., 2021). In addition to serving as the substrate for movement of the wound-activated cells, this ECM can also be a sink for cytokines and growth factors that impact both cell movement and cell fate (Schultz and Wysocki 2009; Wilgus 2012; Frangogiannis 2017). Induction of many of the components of this provisional matrix post-wounding, including collagen I, fibronectin and HA, have been linked to activation of TGFβ (Roberts et al., 1986; Ignotz et al., 1987; Albeiroti et al., 2015). These matrix proteins provide ligands for receptors expressed on the cell surface that participate directly in cell adhesion and in the transmission of signals from the matrix that promote cell migration. In the post-wounding microenvironment, fibronectin, collagen I, HA and TGFβ are all also linked to promoting fibrosis, a pathological outcome to their function in wound repair (Leask and Abraham 2004; Albeiroti et al., 2015; Walraven and Hinz 2018). The post-cataract surgery fibrotic disease PCO is characterized by both cell migration onto the cell-denuded posterior lens capsule and the subsequent appearance of myofibroblasts (Spalton 1999; Wormstone et al., 2009; Wormstone and Eldred 2016; Walker et al., 2018; Wormstone et al., 2021). Collagen I, fibronectin and TGFβ have been linked to fibrosis and fibrotic PCO (Uitto et al., 1982; Serini et al., 1998; Gabbiani 2003; Muro et al., 2008; Herrera et al., 2018; Basta et al., 2021). However, the only evidence that HA may play a role in induction of fibrosis following lens wounding comes from a study with a canine *ex vivo* cataract surgery model where the inclusion of exogenous HA promoted fibrotic PCO (Chandler et al., 2012). Our findings now showed that HA is produced in response to cataract surgery wounding and that its presence in the lens wound environment promotes cell migration and also creates conditions permissive to transitioning the cells involved in lens wound repair to a myofibroblast phenotype.

These findings support developing a therapeutic approach targeting HA production to mitigate the development of the lens fibrotic disease PCO. The use of a small molecular inhibitor to block HA production at the time of cataract surgery, applied directly to the post-cataract surgery lens capsular bag is expected to have limited effects on surrounding eye tissues. The inhibitor used in our study, 4-MU, blocks global HA synthesis by depleting cellular pools of the HAS substrate UDP- glucuronic acid (UDP-GlcUA), which is required for HA synthesis (Kakizaki et al., 2004). 4-MU also was shown to decrease HAS mRNA levels (Kultti et al., 2009). Therefore, treatment with 4-MU will interfere with the function of all three HA synthases, HAS1, HAS2 and HAS3, with the potential to impact both fibrotic and regenerative repair. A better approach would be to identify and target the specific injury activated HAS(s) lead to the lens fibrotic phenotype post-cataract surgery wounding. A likely candidate is HAS2, which is linked to driving fibrosis in other tissues, such as the liver and lung (Li et al., 2016; Yang et al., 2019). The *ex vivo* post-cataract surgery model will provide an ideal reductionist model for future studies in which to determine the function of individual HASs in promoting the fibrotic outcome to cataract surgery wounding.

In studies with the *ex vivo* chick mock cataract surgery explant cultures and human post-cataract surgery explant cultures we now identify the mesenchymal leader cells that rapidly populate the wound edges of the explant and acquire a myofibroblast phenotype as tissue resident immune cells (Menko et al., 2021). Our earlier studies demonstrated that this mesenchymal leader cell population directs the wounded lens epithelium to migrate off the capsule onto and across the surrounding tissue culture substrate (Walker et al., 2010; Menko et al., 2014; Walker et al., 2015; Bleaken et al., 2016), and that their acquisition of myofibroblast phenotype continues to expand over time (Walker et al., 2018). We show that the properties of these resident immune cells includes expression of CD45, MHCII, and the HA receptor CD44 (Menko et al., 2021; Walker and Menko 2021). The interaction between CD44, a hallmark of the leader cell population, and HA is considered a driving force in the recruitment and migration of immune cells (McDonald and Kubes 2015; Nagy et al., 2019).

Studies of the cataract surgery response in CD44 knockout mice shows that the development of fibrotic PCO is not impaired in the absence of this HA receptor (Desai et al., 2010). Our new findings suggest that this role is played by the HA receptor RHAMM. Its pattern of localization in the mesenchymal leader cells post-cataract surgery wounding to lamellipodia processes extended at the leading edge, and along actin stress fibers are consistent with functions in leader cell migration, transition to a myofibroblast, and myofibroblast persistence in fibrosis. Since HA signaling through RHAMM can mediate the induction of cell migration by TGFβ (Samuel et al., 1993), it is possible that RHAMM function is coordinated with TGFβ in promoting a fibrotic outcome. The intracellular population of RHAMM have distinct, and important functions that include roles as a transcriptional regulator (Meier et al., 2014) and in its association with different elements of the cytoskeleton (Assmann et al., 1999). In mitotic cells, RHAMM is a stabilizer of the spindle poles and there is evidence that of a stabilizing role along microtubules and actin filaments (Maxwell et al., 2003). While we find no evidence of a microtubule association in the mesenchymal leader cells post-cataract surgery wounding, we found that RHAMM is localized along the αSMA+ stress fibers of myofibroblasts during the later culture times of our study. A recent study shows that Hyaluronidase-2 (HYAL2) localizes along F-actin rich stress fibers and associates with αSMA in TGFβ-induced myofibroblasts (Midgley et al., 2020). In this study, HYAL-2 is shown to interact with and activate RhoA to regulate myofibroblast migration, contraction, and expression of pro-fibrotic genes such as FN and collagen I (Midgley et al., 2020). The initial localization of RHAMM to lamellipodial extensions of the mesenchymal cells that locate to the leading-edge post-wounding in our studies suggests that RHAMM may be concentrated at integrin focal adhesion complexes, sites where active Rho/ROCK signaling involved in inducing the assembly of both the focal adhesions and the actin stress fibers that directly link to them. We speculate that RHAMM localization to these sites may play a role in the initial formation of actin stress fibers, and the mechanotransduction signaling involved in acquisition of a myofibroblast phenotype. Furthermore, localization of RHAMM along αSMA+ stress fibers formed after the transition of the leader cells to myofibroblasts may function in a similar manner to HYAL2 to regulate their contractile, pro-fibrotic functions. Rho-Rho kinase signaling is critical to the acquisition of a myofibroblast phenotype in the lens (Korol et al., 2016) and for capsule contraction linked to fibrosis in a mouse lens injury model (Ichikawa et al., 2020). Our new findings that RHAMM localizes initially to leader cells in regions rich in integrin focal adhesion complexes and then along the αSMA+ contractile machinery after their transition to myofibroblasts open promising new areas of study for investigating whether there is a functional link between RHAMM and Rho-ROCK signaling in response to cataract surgery wounding. RHAMM’s distinct extracellular and intracellular subcellular patterns observed with the *ex vivo* post-cataract surgery cultures suggest that RHAMM plays multiple location-specific functions to mediate the fibrotic outcome to cataract surgery wounding.

Our PLA studies provide the first evidence that the localization of RHAMM to the lamellipodial processes of mesenchymal leader cells post-wounding reflects its presence in a complex together with both HA and vimentin. Vimentin is an intermediate filament protein with essential functions in both cell migration and wound closure (SundarRaj et al., 1992; Eckes et al., 1998; Eckes et al., 2000; Mendez et al., 2010; Menko et al., 2014). While it is best known as a cytoskeletal filamentous network that provides cells with resistance to mechanical stresses, non-filamentous forms of vimentin have been identified with both intracellular and extracellular functions (Mor-Vaknin et al., 2003; Teshigawara et al., 2013; Menko et al., 2014; Shigyo et al., 2015; Shigyo and Tohda 2016; Walker et al., 2018). The extracellular form of vimentin has been linked to the activation of latent TGFβ (Nishida et al., 2009). Our previous studies show that extracellular vimentin is produced in response to cataract surgery wounding and that an extracellular, cell surface-linked, population of vimentin is involved in signaling the transition of the mesenchymal leader cells to a myofibroblast phenotype (Walker et al., 2018). The *ex vivo* lens-wound explant model closely parallels the complexity of typical wound environments, including the organization of an HA-containing provisional matrix that is formed to promote cell migration and close the wound that also can induce repair-modulating wound-response cells to acquire a myofibroblast phenotype. Our discoveries, including that of an HA/extracellular vimentin/RHAMM axis that is likely linked to both cell migration and the transition of mesenchymal leader cells to myofibroblasts provides a previously unknown key to the mysteries of the antithetical outcomes of healing and fibrosis in the wound environment.

## Data Availability

The original contributions presented in the study are included in the article/Supplementary Material, further inquiries can be directed to the corresponding author.
