# Supplementary figures and images for: The Pro-fibrotic Response of Mesenchymal Leader Cells to Lens Wounding Involves Hyaluronic Acid, Its Receptor RHAMM, and Vimentin
Source: Front Cell Dev Biol. 2022 Mar 21;10:862423. doi: 10.3389/fcell.2022.862423 (PMC8977891; doi:10.3389/fcell.2022.862423)

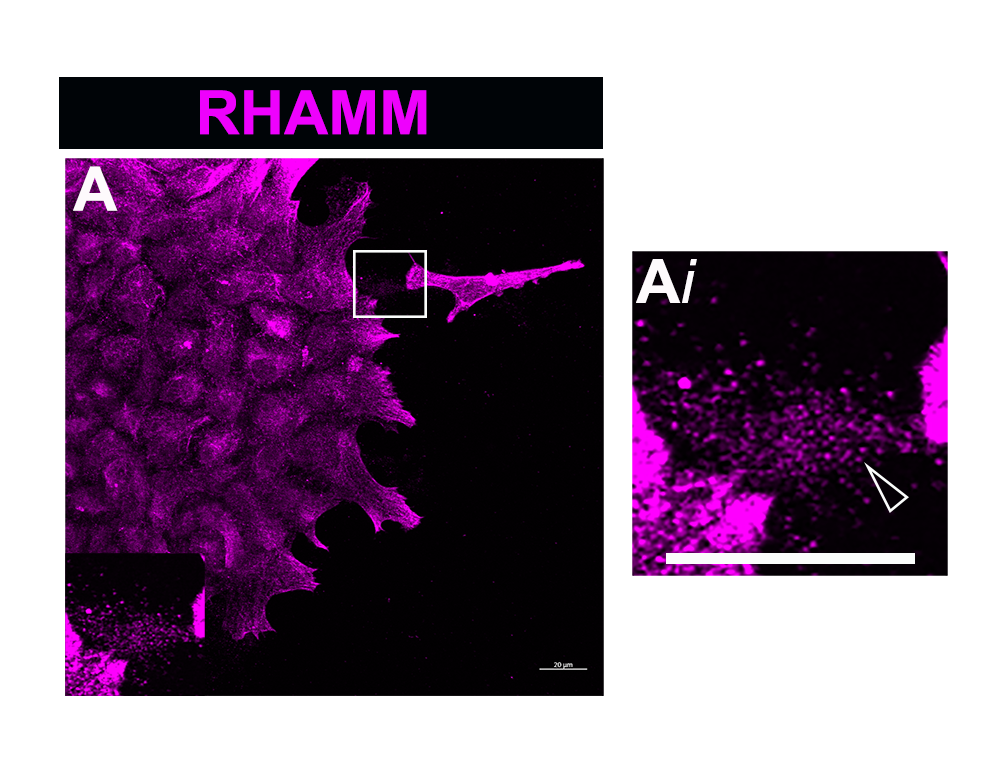

Supplement: Supplementary file 1 [file Image2.TIF]

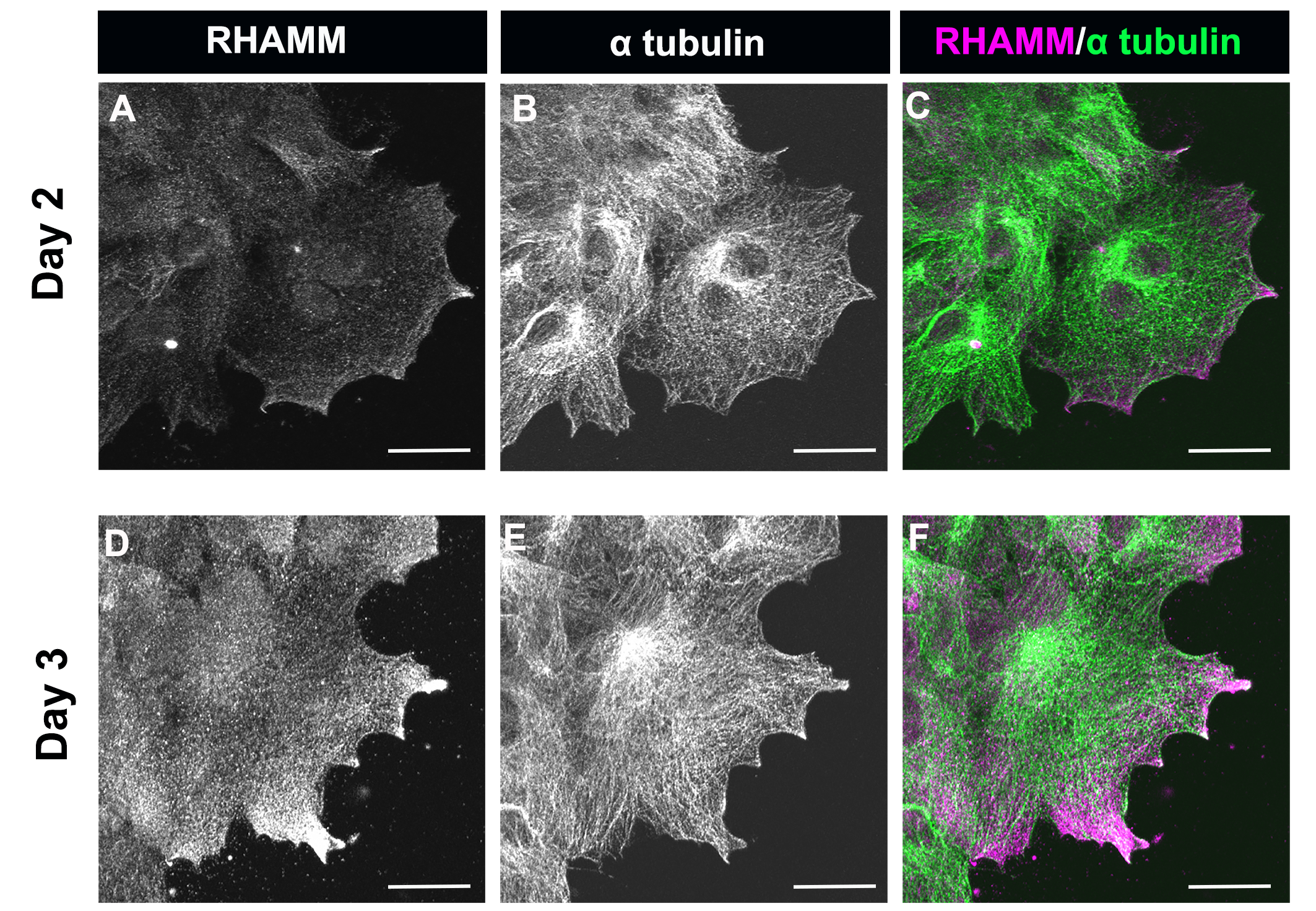

Supplement: Supplementary file 2 [file Image1.TIF]
