# Supplementary material for: The Pro-fibrotic Response of Mesenchymal Leader Cells to Lens Wounding Involves Hyaluronic Acid, Its Receptor RHAMM, and Vimentin
Source: Front Cell Dev Biol. 2022 Mar 21;10:862423. doi: 10.3389/fcell.2022.862423 (PMC8977891; doi:10.3389/fcell.2022.862423)
Supplement: Supplementary file 5 [file DataSheet1.docx]

**Supplemental Figure 1: Co-immunolabeling of RHAMM and tubulin at the leading edge of the ECZ.** Ex vivo lens post-wounding explant cultures at (A-C) D2 and (D-F) D3 co-immunolabeled for (A,D) RHAMM and (B,E) α-tubulin, imaged by confocal microscopy at the leading edge of the ECZ, and (C,F) shown as merged images. No co-localization was detected between RHAMM and the extensive microtubule network of these cells. Images are presented as projections of collected confocal z-stacks with each optical plane at .49 µm. Magnification bars= 20µm.

**Supplemental Figure 2: RHAMM deposited on the substrate by migrating cells**. Ex vivo post-cataract surgery explant culture immunolabeled for RHAMM at D2 and imaged by confocal microscopy in a region where an individual mesenchymal cell has migrated away from the leading edge of the ECZ. The boxed region in (A) is shown at higher magnification and greater intensity in (A*i*), which highlights punctate RHAMM staining along the substrate surface between the leading edge and the cell that has migrated beyond the leading edge (open arrowhead). Images are presented as a projection of a collected confocal z-stack with each optical plane at .49 µm. Magnification bars= 20µm.

**Supplemental Movies 1 and 2: Blocking HA synthesis inhibits leader cell extension and migration across the tissue substrate.** Ex vivo post-cataract surgery explants were treated in culture from D1-D2 post-wounding with the HA synthesis inhibitor 4-MU (400µM) (Movie 2) or its vehicle DMSO (Movie 1). Time-lapse movies of the ECZ were taken from day 1 through day 2, revealing that 4-MU treatment blocked leader cell extension and cell migration in the ECZ region compared to vehicle controls.
